# Identification of biological targets of therapeutic intervention for clear cell renal cell carcinoma based on bioinformatics approach

**DOI:** 10.1186/s12935-016-0291-8

**Published:** 2016-03-03

**Authors:** Yongsheng Chen, Lichen Teng, Wenhua Liu, Yan Cao, Dexin Ding, Wentao Wang, Hui Chen, Changfu Li, Ruihua An

**Affiliations:** Department of Urology, Harbin Medical University Cancer Hospital, Harbin, 150040 Heilongjiang Province China; Intensive Care Unit (ICU) Department, The Second Affiliated Hospital of Harbin Medical University, Harbin, 150086 Province Heilongjiang China; Department of Urology, The First Affiliated Hospital of Harbin Medical University, No.31 Youzheng Street, Harbin, 150001 Province Heilongjiang China

**Keywords:** Clear cell renal cell carcinoma, MicroRNAs, Differentially expressed genes, Protein–protein interaction

## Abstract

**Background:**

We aimed to discover the potential microRNA (miRNA) targets and to explore the underlying molecular mechanisms of clear cell renal cell carcinoma (ccRCC).

**Methods:**

Microarray data of GSE16441 was downloaded from Gene Expression Omnibus database. Differentially expressed genes (DEGs) and differentially expressed miRNAs between ccRCC tumors and matched non-tumor samples were analyzed. Target genes of differentially expressed miRNAs were screened. Besides, functional enrichment analysis of DEGs was performed, followed by protein–protein interaction (PPI) network construction and sub-module analysis. Finally, the integrated miRNA-DEGs network was constructed.

**Results:**

A total of 1758 up- and 2465 down-regulated DEGs were identified. Moreover, 15 up- and 12 down-regulated differentially expressed miRNAs were screened. The up-regulated DEGs were significantly enriched in pathways such as cell adhesion molecules and focal adhesion. Besides, the down-regulated DEGs were enriched in oxidative phosphorylation, and citrate cycle (TCA cycle). Moreover, eight sub-modules of PPI network were obtained. Totally, eight down-regulated miRNAs were identified to significantly regulate the DEGs and miRNA-200c that could regulate collagen, type V, alpha 2 (COL5A2) as well as COL5A3 was found to be the most significant. Additionally, 10 up-regulated miRNAs were identified to be significantly associated with the DEGs. Thereinto, miRNA-15a that could regulate ATPase, H^+^ transporting, lysosomal 21 kDa, V0 subunit b (ATP6V0B) and miRNA-155 were found to be the most significant.

**Conclusions:**

miRNA-200c that could regulate COL5A2 and COL5A3, miRNA-15a that could regulate ATP6V0B and miRNA-155 may play key roles in ccRCC progression. These miRNAs may be potential targets for ccRCC treatment.

## Background

Renal cell carcinoma (RCC) represents the most lethal genitourinary malignancy and about 90 % of all kidney tumors are RCC [1]. Clear cell RCC (ccRCC) is the most common subtype of RCC, accounting for about 75 % of cases [2]. Because of being generally asymptomatic, one-third of ccRCC are already metastatic at initial diagnosis, leading to a high mortality rate up to 95 % [3]. However, the benefit of surgical, chemotherapeutic and radiological approaches for ccRCC is limited [3, 4]. Thus, it is of great importance to gain a better understanding of the pathogenesis of ccRCC, which may facilitate the development of effective biomarkers and novel, targeted therapeutic strategies.

Major efforts have been carried out to explore the underlying etiology and molecular mechanisms of ccRCC. Cigarette smoking, obesity, hypertension, and acquired cystic kidney disease were shown to be associated with renal cancer [5]. There is ample evidence indicating that the most frequent event in ccRCC is inactivation or mutations in the von-Hippel Lindau (VHL) tumor suppressor gene, which induces the expression of the hypoxia inducible factors (HIF)1α and HIF2α [6, 7]. Two of the other most commonly mutated genes in ccRCC are PBRM1 (about 40–50 %) and BAP1 (10–15 %) [8]. In addition, extracellular signal-regulated kinase 5 (ERK5) is demonstrated to mediate angiogenesis, proliferation, and tumor aggressiveness in ccRCC [9]. By contrast, microRNAs (miRNAs), a group of small non-coding RNAs (21–25 nucleotides) that regulate expression of target genes at the post-transcriptional level, are crucial for gene-regulatory networks which play an important role in different biological processes, including metabolic disorder, tissue injury, and oxidative stress [10, 11]. Aberrant alterations in miRNA expression have been shown to be related to human malignancies [12, 13]. Numerous miRNAs have also been identified in ccRCC in which some were demonstrated to function as oncogenes, while others were identified to play tumor suppressor roles [14, 15]. For instance, Mathew et al. have demonstrated that the suppression of miR-30c-2-3p as well as miR-30a-3p increases HIF2α levels in ccRCC, promoting angiogenesis, cellular proliferation, and tumor growth [16]. In addition, Liu et al. have suggested miR-23b* was up-regulated in renal cancer as an important regulator of proline oxidase [17]. Additionally, a number of array platform-based studies recently indicate that a considerable number of miRNAs are dysregulated in ccRCC [18]. Although the results of previous studies may not be consistent, all the data indicate that dysregulated miRNAs may play pivotal roles in the pathogenesis of ccRCC. However, the present knowledge is still poorly understood.

In the current study, we employed the bioinformatics methods to identify the differentially expressed genes (DEGs) and differentially expressed miRNAs between ccRCC tumors and corresponding non-tumor samples. Target genes of differentially expressed miRNAs were screened. Then, functional enrichment analysis of DEGs was performed, followed by protein–protein interaction (PPI) network construction and sub-module analysis. Finally, an integrated miRNA-DEG network was constructed. We intended to give a systematic perspective on understanding the molecular mechanism and to investigate more potential therapeutic targets for ccRCC.

## Methods

### Data collection

The expression profile of GSE16441, deposited by Liu et al. [19] was downloaded from the National Center of Biotechnology Information (NCBI) Gene Expression Omnibus (GEO) database [20] (http://www.ncbi.nlm.nih.gov/geo/). The GSE16441 dataset is composed of gene expression microarray data and miRNA microarray data. There were 34 samples (GSM413237-GSM413270) including 17 ccRCC tumors and 17 adjacent normal kidney tissue samples in the gene expression microarray data. The platform is Agilent-014850 Whole Human Genome Microarray 4x44 K G4112F (Probe Name version). Moreover, 34 samples from 17 ccRCC tumors and 17 corresponding non-tumor samples were obtained using a platform of Agilent Human miRNA Microarray Rel12.0 for miRNA expression analysis. In this study, all the samples were used for the follow-up analysis.

### Data preprocessing and identification of DEGs and differentially expressed miRNAs

Data in CEL format were downloaded and converted into expression values, followed by background correction using Normexp algorithm [21]. Then, data were normalized by the Loess method [22]. Subsequently, the linear models for microarray data (Limma) package [23] from Bioconductor was applied to screen DEGs between RCC tumors and 17 corresponding non-tumor samples. The associated P values were adjusted to false discovery rates (FDR) based on the Benjamini and Hochberg method [24]. FDR <0.05 and |log FC| >1 were selected as the cutoff criterion for DEGs screening.

Similarly, with respect to the screening of differentially expressed miRNAs, Normexp method was used to perform the background correction. Then, data were normalized by the Quantile method [25]. Subsequently, Limma package from Bioconductor was applied to identify differentially expressed miRNAs between ccRCC tumors and 17 corresponding non-tumor samples. FDR <0.05 and |log FC|> 1 were defined as the cutoff criterion for the screening of differentially expressed miRNAs.

### The analysis of target genes of differentially expressed miRNAs

The genes targeted by the miRNAs were screened based on seven databases (miRanda, MirTarget2, PicTar, PITA, TargetScan, miRecords as well as MirWalk). The combination of the seven databases was the criteria for target gene databases. Then, these target genes were further processed to investigate the more potential miRNA-DEG pairs by using the Lasso regression method, which was proposed to identify miRNA–mRNA targeting relationships with considerable advantages in both sensitivity and specificity [26]. The criteria was set as select.score >90 and FDR <0.05.

### Gene ontology (GO) and pathway enrichment analysis of DEGs

GO analysis has been used as functional annotation of large-scale genes frequently [27]. Database for Annotation, Visualization and Integrated Discovery (DAVID) [28] is a tool providing a comprehensive set of functional annotation. In this study, DAVID was utilized for GO enrichment analysis to identify the significantly enriched biological process (BP) terms of DEGs, with P value <0.05.

Kyoto Encyclopedia of Genes and Genomes (KEGG) database includes all kinds of biochemistry pathways [29]. In this study, DAVID was also applied for KEGG pathway enrichment analysis of DEGs. P < 0.05 was chosen as the cutoff value.

### PPI network construction and significant sub-module extraction

The Search Tool for the Retrieval of Interacting Genes (STRING) database offers both experimental and predicted interaction information [30]. In this study, STRING database was applied to identify functional interactions between DEGs and a PPI network was then constructed, which was visualized with Cytoscape software [31]. The combined score >0.9 was selected as the threshold.

In order to further analyze the more significant interactions from the PPI network, functional sub-modules were extracted using ClusterOne [32].

### Construction of integrated miRNA-DEG network

Based on the results of the identified potential target genes of differentially expressed miRNAs from databases and results of the significant sub-modules of PPI network, the integrated miRNA-DEG network was constructed.

## Results

### Identification of DEGs and differentially expressed miRNAs

Totally, 1758 up-regulated DEGs (corresponding to 1804 up-regulated transcripts), and 2465 down-regulated DEGs (corresponding to 2566 down-regulated transcripts) were screened out in ccRCC samples compared with matched non-tumor samples. Moreover, a total of 15 up-regulated and 12 down-regulated differentially expressed miRNAs were identified in ccRCC samples compared with matched control samples.

### The analysis of target genes of miRNAs

According to the criteria of select.score >90 and FDR <0.05, target genes of the up- and down-regulated miRNAs were obtained. Number of target genes for the up- and down-regulated miRNAs is listed in Tables 1, 2 respectively.

**Table 1:** The number of target genes of up-regulated miRNAs

miRNA	The number of target genes
hsa-miR-106b	303
hsa-miR-126	5
hsa-miR-142-3p	31
hsa-miR-142-5p	66
hsa-miR-144	30
hsa-miR-155	114
hsa-miR-15a	149
hsa-miR-16	70
hsa-miR-21	16
hsa-miR-210	11
hsa-miR-223	32
hsa-miR-27a	126
hsa-miR-34a	134

**Table 2:** The number of target genes of down-regulated miRNAs

miRNA	The number of target genes
hsa-miR-141	21
hsa-miR-199a-5p	29
hsa-miR-200a	63
hsa-miR-200b	22
hsa-miR-200c	134
hsa-miR-204	17
hsa-miR-30a	39
hsa-miR-30d	69
hsa-miR-363	26
hsa-miR-429	20

### GO and pathway enrichment analysis of DEGs

The top 5 GO BP terms of up-regulated DEGs and down-regulated DEGs were shown in Table 3. From the results, we found that the up-regulated DEGs were significantly enriched in immune response (P = 9.10E−18), cell activation (P = 3.82E−13), positive regulation of immune system process (P = 2.70E−12), regulation of cell activation (P = 5.70E−12) and leukocyte activation (P = 1.86E−11). While the down-regulated DEGs mainly participated in oxidation reduction (P = 2.56E−16), generation of precursor metabolites and energy (P = 2.93E−15), ion transport (P = 4.19E−13), hydrogen transport (P = 1.38E−12) and proton transport (P = 3.91E−12).

**Table 3:** The top five enriched gene ontology (GO) terms functional enrichment of the differentially expressed genes

	GO ID	Term	Gene counts	P value
Up-regulated genes	GO:0006955	Immune response	104	9.10E−18
	GO:0001775	Cell activation	54	3.82E−13
	GO:0002684	Positive regulation of immune system process	47	2.70E−12
	GO:0050865	Regulation of cell activation	39	5.70E−12
	GO:0045321	Leukocyte activation	46	1.86E−11
Down-regulated genes	GO:0055114	Oxidation reduction	132	2.56E−16
	GO:0006091	Generation of precursor metabolites and energy	80	2.93E−15
	GO:0006811	Ion transport	141	4.19E−13
	GO:0006818	Hydrogen transport	29	1.38E−12
	GO:0015992	Proton transport	28	3.91E−12

The pathways that were significantly enriched by the up- and down-regulated genes were shown in Tables 4, 5, respectively. The results showed that the up-regulated DEGs were significantly associated with several pathways such as primary immunodeficiency (P = 1.63E−06), cell adhesion molecules (CAMs) (P = 6.66E−06), natural killer cell mediated cytotoxicity (P = 2.13E−04), cytokine–cytokine receptor interaction (P = 3.52E−04). By contrast, the down-regulated DEGs were significantly enriched in oxidative phosphorylation (P = 1.83E−13), valine, leucine and isoleucine degradation (P = 1.31E−07) and citrate cycle (TCA cycle) (P = 4.63E−07).

**Table 4:** Kyoto encyclopedia of genes and genomes (KEGG) pathway analysis of up-regulated differentially expressed genes

Category	Term	Count	P value
KEGG	Primary immunodeficiency	13	1.63E−06
KEGG	Cell adhesion molecules (CAMs)	25	6.66E−06
KEGG	Natural killer cell mediated cytotoxicity	22	2.13E−04
KEGG	Cytokine-cytokine receptor interaction	34	3.52E−04
KEGG	Viral myocarditis	14	8.38E−04
KEGG	Focal adhesion	27	0.001001
KEGG	Antigen processing and presentation	15	0.001245
KEGG	T cell receptor signaling pathway	17	0.002368
KEGG	Allograft rejection	9	0.002374
KEGG	Graft-versus-host disease	9	0.004036

**Table 5:** Kyoto encyclopedia of genes and genomes (KEGG) pathway analysis of down-regulated differentially expressed genes

Category	Term	Count	P value
KEGG	Oxidative phosphorylation	48	1.83E−13
KEGG	Valine, leucine and isoleucine degradation	20	1.31E−07
KEGG	Citrate cycle (TCA cycle)	16	4.63E−07
KEGG	Alzheimer’s disease	42	1.07E−06
KEGG	Arginine and proline metabolism	20	3.97E−06
KEGG	Parkinson’s disease	34	6.95E−06
KEGG	Aldosterone-regulated sodium reabsorption	16	3.16E−05
KEGG	Vibrio cholerae infection	18	1.45E−04
KEGG	Cardiac muscle contraction	22	1.70E−04
KEGG	Alanine, aspartate and glutamate metabolism	12	4.94E−04

### PPI network construction and sub-modules mining

**Fig. 1:**
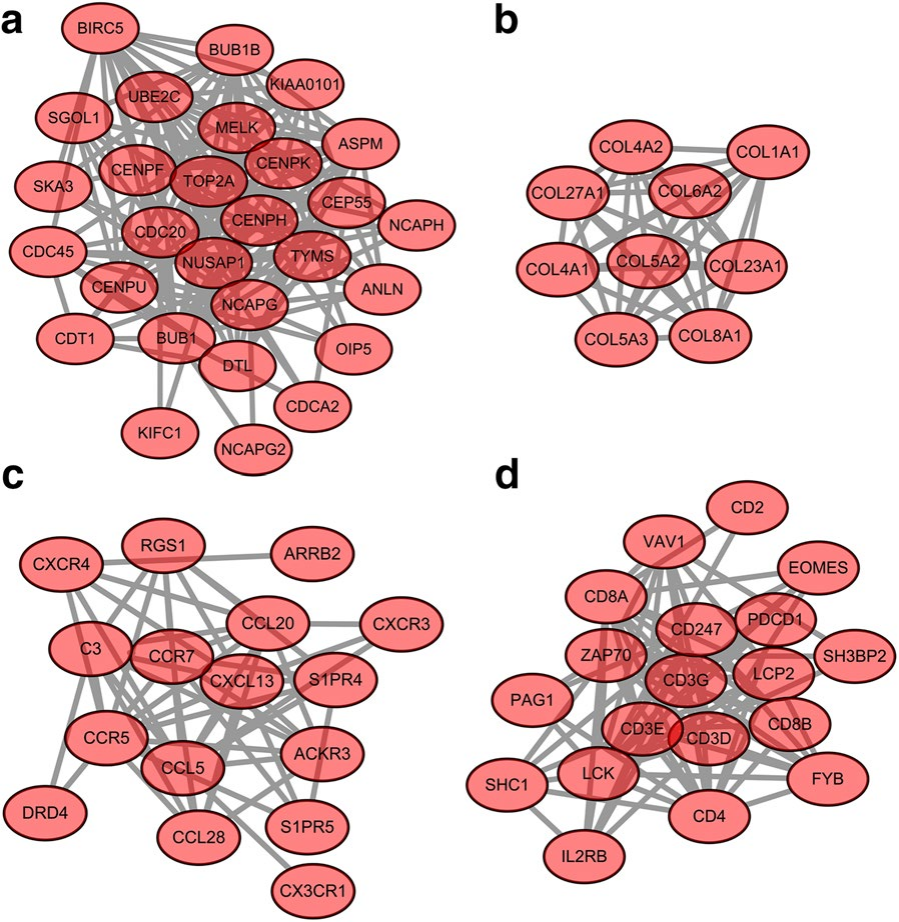
The four significant sub-modules of up-regulated genes (sub-module **a**, **b**, **c**, **d**) in PPI network. The *red oval* nodes denote the up-regulated genes

Totally, four most significant sub-modules of up-regulated DEGs (sub-module a, b, c, and d) were extracted from PPI network as shown in Fig. 1. Sub-module a including 28 nodes and 162 edges was enriched in cell cycle. Sub-module b containing 9 nodes and 36 edges was related with ECM-receptor interaction. Sub-module c with 16 nodes and 54 edges was enriched in chemokine signaling pathway. Besides, 19 nodes combined with 89 edges made up sub-module d which was enriched in T cell receptor signaling pathway.

**Fig. 2:**
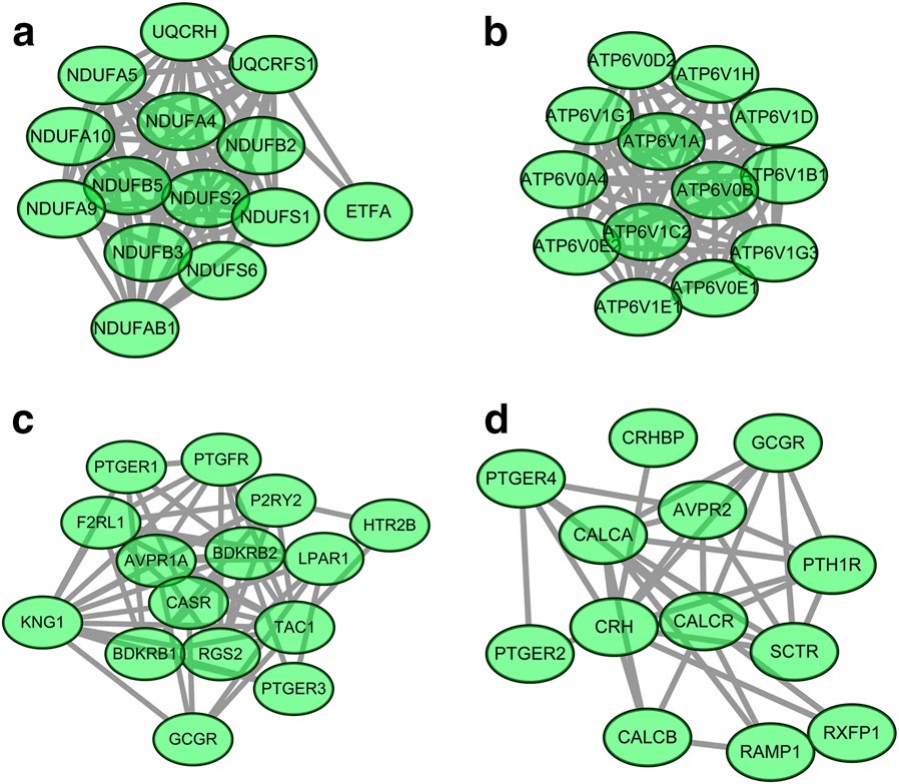
The four significant sub-modules of down-regulated genes (sub-module **a**, **b**, **c**, **d**) in PPI network. The *green oval* nodes denote the down-regulated genes

Additionally, a total of four significant sub-modules of down-regulated genes (sub-module a, b, c, d) were achieved as shown in Fig. 2. Sub-module a including 14 nodes and 80 edges was enriched in oxidative phosphorylation. Sub-module b with 13 nodes and 78 edges was involved in oxidative phosphorylation. Sub-module c with 15 nodes and 56 edges was enriched in neuroactive ligand-receptor interaction. Moreover, 15 nodes combined with 56 edges made up sub-module d which was related with neuroactive ligand-receptor interaction.

### Construction of integrated miRNA-DEG network

**Fig. 3:**
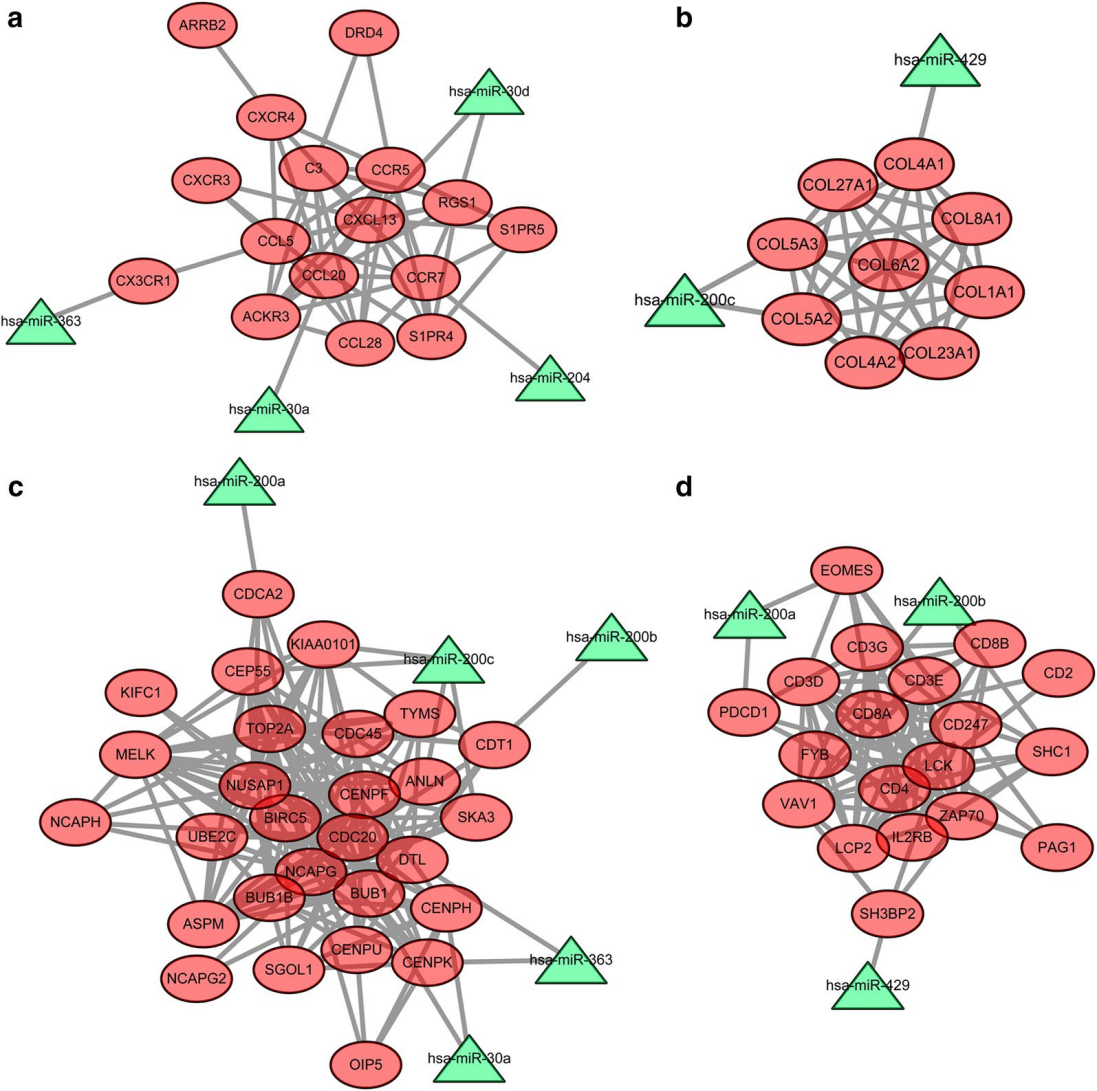
The network of up-regulated DEGs in four sub-modules (**a**, **b**, **c**, **d**) and their related miRNAs. The *red oval* nodes are the up-regulated DEGs, and *green triangle* nodes are the down-regulated miRNAs

**Fig. 4:**
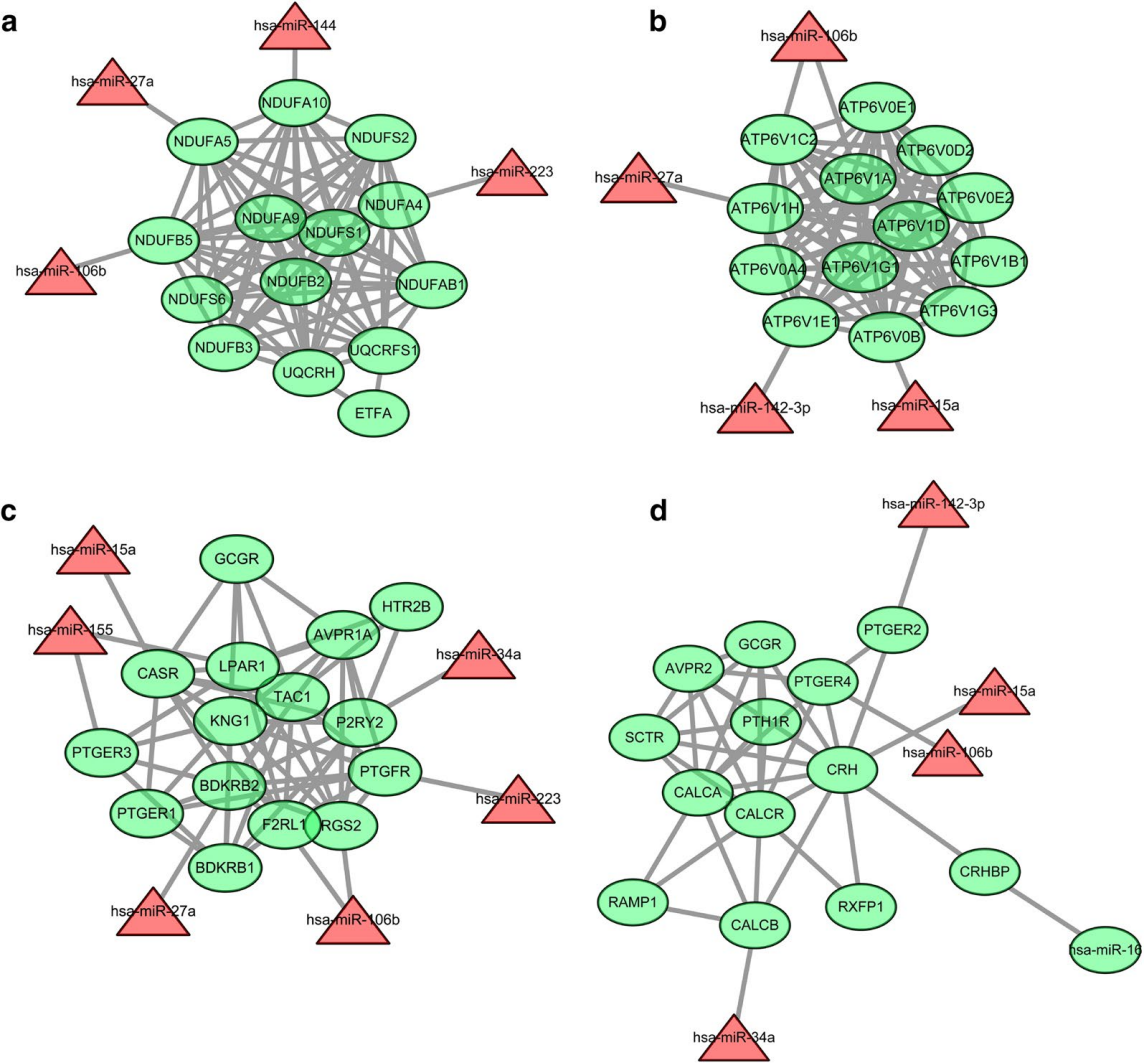
The network of down-regulated DEGs in four sub-modules (**a**, **b**, **c**, **d**) and their related miRNAs. The *green oval* nodes are the down-regulated DEGs, and *red triangle* nodes are the up-regulated miRNAs

The miRNA-DEG network was constructed by integrating significant DEGs in sub-modules and potential miRNAs-DEG pairs. Up-regulated DEGs in the four identified sub-modules and their related miRNAs (down-regulated) were shown in Fig. 3. Moreover, down-regulated DEGs in the four identified sub-modules and their related miRNAs (up-regulated) were shown in Fig. 4.

## Discussion

ccRCC is the most common histological subtype of RCC that occurs in adults and associated with worse prognosis [33]. In this study, we applied bioinformatics method to predict the potential miRNA targets for the treatment of ccRCC progression. Our results suggested that 1758 up- and 2465 down-regulated DEGs were screened out in ccRCC samples. Moreover, a total of 15 up- and 12 down-regulated differentially expressed miRNAs were identified. The up-regulated DEGs were enriched in significant pathways such as CAMs and focal adhesion. Besides, the down-regulated DEGs were significantly associated with oxidative phosphorylation, and TCA cycle. Several significant differentially expressed miRNAs were identified and miRNA-200 family was found to be the most significant.

miRNA-200 family includes miRNA-200a, miRNA-200b, miRNA-200c, miRNA-429, and miRNA-141 [34]. In the present work, miR-200a, miR-200b, miR-200c and miR-429 were identified from the network of DEGs and their related miRNAs. Additionally, these miRNAs were down-regulated in ccRCC. As previously reported, the members of the miRNA-200 family (especially miR-200c and miR-141) play an outstanding role as metastasis suppressor genes via inhibiting the expression of zinc finger E-box binding homeobox 1 (ZEB1) [35, 36]. Under-expression of miRNA-200 family members is correlated with renal cancer [37]. Moreover, the elevation of collagens and fibronectin in obstructed kidneys can be repressed by the injection of miR-200b [38]. Previous reports exhibited that the increased level of type V collagen has been detected in human breast cancer and in mouse skin tumors [39, 40]. In the current study, we found that under-expression of miR-200c targeted and up-regulated the level of collagen, type V, alpha 2 (COL5A2) and COL5A3. Besides, we found that the up-regulated DEGs were significantly associated with CAMs and focal adhesion (Table 4). Amounting evidence has demonstrated that collagens and fibronectin contributes to cell adhesion dynamics and cell migration which are significantly concerned with tumor metastasis [41, 42]. Taken together, we infer that miRNA-200 family may regulate several genes such as COL5A2 and play a critical role in the progression in ccRCC through participating in cell adhesion and migration. The members of the miRNA-200 family, especially miRNA-200c, may constitute novel therapeutic targets in ccRCC and further experimental verifications are needed.

Out of the highly up-regulated miRNAs, miRNA-15a was a member of the miRNA-15 precursor family including miRNA-15a, miRNA-15b, miRNA-16-1, miRNA-16-2, miRNA-195 and miRNA-497. Metabolic activities for energy in normal cells depend on mitochondrial oxidative phosphorylation (OXPHOS) primarily, but OXPHOS capacity is decreased in various cancer cells [43]. The process of OXPHOS needs oxygen. Oxygen deficiency leads to the inhibition of OXPHOS, mainly mediated by the HIF-1 [44]. HIF is a master regulator of RCC metabolism [45]. Moreover, the miRNA-15a can regulate oxygen consumption and adenosine triphosphate (ATP) production via targeting uncoupling-protein 2 [46]. The low ATP synthase often observed in ccRCC [47]. In the present study, the up-regulated miRNA-15a was identified to regulate the ATPase, H+ transporting, lysosomal 21 kDa, V0 subunit b (ATP6V0B) gene which was significantly enriched in the pathway OXPHOS. Moreover, pathway enrichment analysis of down-regulated genes showed these genes were significantly linked with oxidative phosphorylation (Table 5). Hence, we infer that miRNA-15a may play a crucial role in the pathogenesis of ccRCC via being involved in oxidative phosphorylation. miRNA-15a may provide a novel therapeutic target in ccRCC and further experiments are needed to verify this finding.

Another up-regulated miRNA in human cancers is miRNA-155, which is described as an oncogene [48]. Previous studies demonstrated that miRNA-155 was up-regulated in various kinds of human malignancy, including breast cancer, non-small cell lung cancer and lymphomas [49–51]. Recent studies have also exhibited the up-regulation of miRNA-155 levels in renal cancers [52, 53]. In accordance with the previous studies, our results found that miRNA-155 was up-regulated in RCC samples. Moreover, the inhibition of miRNA-155 expression induced apoptosis, suppressed proliferation and migration in renal cancer cells [54]. Collectively, our results further confirmed that therapy targeting miRNA-155 inhibition may be an effective approach for ccRCC treatment.

However, our study has several limitations. For instance, analysis of the other data in similar topic with larger samples may be beneficial to cross-check of the results in our study. Besides, there was a lack of experimental verifications. We intend to perform further experimental verifications in our future studies using different methods as the other study performed [14, 19], such as immunohistochemistry, and quantitative RT-PCR.

## Conclusions

In summary, the identified DEGs and their related miRNAs, especially miRNA-200c and its target genes COL5A2 as well as COL5A3, miRNA-15a and its target genes ATP6V0B and miRNA-155 may play key roles in the progression of ccRCC, and these may be useful biomarkers for predicting tumor metastasis and therapeutic targets for the treatment of ccRCC. However, further experiments are needed to validate the effects and mechanisms of miRNA-200c, miRNA-15a and miRNA-155 in ccRCC.

## Abbreviations

ATP: adenosine triphosphate
CAMs: cell adhesion molecules
ccRCC: clear cell renal cell carcinoma
DEGs: differentially expressed genes
ERK5: extracellular signal-regulated kinase 5
GEO: gene expression omnibus
NCBI: National Center of Biotechnology Information
OXPHOS: oxidative phosphorylation
PBRM1: polybromo 1
PPI: protein–protein interaction
VHL: von-Hippel Lindau
ZEB1: zinc finger E-box binding homeobox 1
